# Association between sedentary time and plasma leptin levels in middle-aged and older adult population in Taiwan: A community-based, cross-sectional study

**DOI:** 10.3389/fcvm.2022.1057497

**Published:** 2023-01-09

**Authors:** Yu-Lin Shih, Yun-Hsiang Shih, Tzu-Cheng Huang, Chin-Chuan Shih, Jau-Yuan Chen

**Affiliations:** ^1^Department of Family Medicine, Chang Gung Memorial Hospital, Taoyuan, Taiwan; ^2^School of Medicine, Medical University of Lublin, Lublin, Poland; ^3^General Administrative Department, United Safety Medical Group, New Taipei City, Taiwan; ^4^College of Medicine, Chang Gung University, Taoyuan, Taiwan

**Keywords:** sitting time, sedentary lifestyle, biomarker, leptin, obesity, cardiometabolic diseases

## Abstract

**Background:**

Association of sedentary behavior and plasma leptin levels is a popular topic in recent research. Aged populations often suffer from cardiometabolic diseases, and leptin is considered a novel marker for many cardiometabolic diseases. To further explore this topic, our research investigates the relationship between sedentary time and serum leptin levels in middle-aged and older populations.

**Methods:**

A total of 396 middle-aged and older adult Taiwanese participants were included in this study. We recorded their self-reported sitting time as sedentary time. Participants were categorized into low leptin, medium leptin group, and high leptin groups according to the tertile of serum leptin level in the study. We also analyzed the anthropometric and cardiometabolic parameters between the three groups. Spearman's correlation coefficient was used to analyze the correlation between leptin level, sedentary time, and other cardiometabolic risk factors. The relationsip between leptin and sedentary time was also shown in a scatter plot. Multivariate linear regression was performed to determine the association between serum leptin levels and sedentary time after adjusting for age, sex, alcohol consumption, smoking, triglycerides, body mass index (BMI), fasting plasma glucose, systolic blood pressure, uric acid, creatinine, and alanine transaminase (ALT).

**Results:**

In our study, data from a total of 396 participants were analyzed. The average age of participants was 64.75 (±8.75) years, and ~41.4% were male. A longer period of sedentary time was observed in the high leptin group. A positive correlation was found between serum leptin level and sedentary time in Spearman's correlation, in all BMI groups. Serum leptin levels were positively associated with sedentary time (B = 0.603, *p* = 0.016) in the multivariate linear regression after adjusting for age, sex, alcohol consumption, smoking, triglycerides, BMI, fasting plasma glucose, systolic blood pressure, uric acid, creatinine, and ALT.

**Conclusion:**

Prolonged sedentary time can be an independent risk factor for high serum leptin levels, and high leptin levels can be a novel marker in future healthcare to screen the individual with prolonged sedentary time. Furthermore, based on our study, future research can further explore the relationship between leptin levels and health promotion, especially decreasing sedentary time in the middle-aged and elder population, which is vulnerable to cardiometabolic diseases.

## 1. Introduction

Lifestyles have changed dramatically in the modern era as technology provides solutions to replace human labor. People are freed from heavy tasks, and less physical activity brings new health problems (1). According to the World Health Organization, physical inactivity contributes to ~2 million deaths annually, and 60–85% of the population in developed and developing countries leads a sedentary lifestyle (2). Previous studies have shown that a sedentary lifestyle leads to chronic diseases such as metabolic disorders, cardiovascular disease, cancer, and depression (3). A sedentary lifestyle also increases overall mortality in the population (4). Among all sedentary behaviors, sitting time can be a useful measure and has been used in many studies to assess the sedentary lifestyle (5–7).

Leptin, a small peptide hormone, is secreted by adipocytes and encoded by the obesity gene (ob) (8, 9). Serum leptin levels positively relate to the amount of adipose tissue in the body (10). In the brain, leptin regulates appetite and energy balance (11). In peripheral tissues, leptin levels control the number of adipocytes and energy balance (12, 13). The main roles of leptin are in energy hemostasis and to function as an anti-obesity hormone (14, 15). Moreover, the pathophysiological functions of leptin have been revealed in previous studies. Leptin resistance is found in many overweight patients and exacerbates their obesity (16). Many cardiometabolic diseases or dysfunctions, such as metabolic syndromes, type 2 diabetes mellitus (DM), or hypertension (HTN), are positively related to serum leptin levels (17, 18). Therefore, high leptin levels are closely related to metabolic diseases in which excessive adipocytes can increase leptin levels (19). However, recent studies have revealed that leptin has other systemic functions besides energy regulation, including neuroendocrine and immunological functions (20, 21). In addition to metabolic disorders, leptin levels also relates to chronic diseases such as cardiovascular disease, chronic kidney disease, mood disorders, and other diseases (22–24). For these reasons, leptin is a potential indicator of health status.

The benefits of intense physical activity are well-known. However, the adverse effects of sedentary behavior, which includes prolonged sitting, need further research. Some studies suggest that prolonged sedentary time has adverse health effects, including type 2 DM, cardiovascular disease, cancer, and high mortality (25). A major study has found that sedentary behavior is responsible for 9% of premature mortality worldwide (9). The study with participants of the Multi-Ethnic Study of Atherosclerosis in the United States showed a positive relationship between leptin levels and sedentary behavior (26). A diabetes prevention program conducted in high-risk individuals in Leicestershire, United Kingdom, indicated a positive relationship between leptin levels and self-reported sitting time, independent of physical activity intensity (27). These previous studies have shown a strong relationship, even independent of intense physical activity. However, aging is a risk factor for cardiometabolic disease (28), and there is a lack of research focused on the relationship between sedentary time and leptin levels in middle-aged and older adult populations which are vulnerable to cardiometabolic diseases. Besiedes, previous research recruited the participants mostly from high-risk populations which are different from community-indwelling populations. Therefore, we want to investigate the relationship between leptin levels and sedentary time in the middle-aged and older adult population in community in Taiwan.

## 2. Methods

### 2.1. Study design and participants

Participants in this cross-sectional, community-based study were recruited through a community health survey project conducted in northern Taiwan in 2019. Participants were selected according to the following inclusion criteria: (1) walking ability, (2) finishing all examinations, (3) finishing the questionnaire, (4) age between 85 and 50 years, and (5) indwell in the community. Some were excluded based on the following exclusion criteria: (1) incomplete examinations or questionnaires or (2) a history of recent heart disease. A total of 396 participants qualified for analysis and were included in our study. An interview was conducted, and participants were asked to complete a questionnaire that included personal information and medical history. We used G^*^power 3.1 to determine the sample size of our study. Considering the linear multiple regression, which we performed in Table 3, we used 0.13, 0.05, 0.8, and 10 as effect size, alpha error, power, and number of predictors, respectively, to calculate the sample size. The estimated total sample size was 118, and the 396 participants in our study provided sufficient statistical power. Written informed consent to participate in the study was obtained before participation in the study. The study was approved by the Chang Gung Medical Foundation Institutional Review Board (No. 201801803B0).

### 2.2. Data collection and measurements

We collected information by questionnaire, including age, sex, alcohol consumption (drinking alcohol more than 2 days per week or not), and current smoking habits (current smoker or not). Alcohol consumption and current smoking habits were both self-reported. We also recorded the respondents' self-reported sitting time as sedentary time through the questionnaire. Information in medical documents such as DM, HTN, and dyslipidemia was also collected. Body mass index (BMI) was calculated using the formula: weight in kilograms divided by the square of height in meters. Diastolic blood pressure (DBP, mmHg) and systolic blood pressure (SBP, mmHg) were measured more than twice after resting. Waist circumference was defined as the measurement midway between the iliac crest and the last rib, in a horizontal plane, while standing. Participants' biochemical laboratory data were analyzed in the Roche model laboratory at Taiwan E&Q Clinical Laboratory using Roche cobas^®^ connection modules (CCM). Laboratory data included triglyceride level (TG, mg/dL), fasting plasma glucose (FPG, mg/dL), low-density lipoprotein (LDL-C, mg/dL), high-density lipoprotein (HDL-C, mg/dL), creatinine (mg/dL), and alanine transaminase (ALT, mg/dL). Leptin level (ng/mL) was analyzed by Enzyme-linked Immunosorbent Assay using Invitrogen™ Human Leptin, and the detection range was 0.024–100,000 ng/mL.

### 2.3. Definition of leptin level and other variables

Based on the tertile of the serum leptin level, we divided the participants into three groups. Participants with a leptin level < 10.8 were placed in the low tertile group, participants with a leptin level from 10.8 to 21.99 were placed in the middle tertile group, and participants with a leptin level ≥ 22.00 were placed in the high tertile group. The criteria for overweight in Taiwan (BMI ≥25 kg/m^2^) was also applied to categorize the participants. HTN was defined as SBP ≥140 mmHg, DBP ≥90 mmHg, or currently being treated for HTN. DM was defined as an FPG ≥126 mg/dL or currently receiving insulin therapy or oral hypoglycemic agents. The definition of dyslipidemia was TG ≥150 mg/dL, total cholesterol ≥200 mg/dL, HDL-C < 40 mg/dL in men or HDL-C < 50 mg/dL in women, and LDL-C ≥130 mg/dL, or use of lipid-lowering medications.

### 2.4. Statistical analysis

Participants were divided into three groups according to leptin levels: low, medium, and high. The normality of continuous data was checked using the Shapiro–Wilk normality test. In Table 1, data that conformed to a normal distribution (sedentary time, age, LDL-C, WC, BMI, SBP, DBP) were presented as mean ± [SD]; data that did not conform to a normal distribution (leptin, FPG, TGs, HDL-C, ALT, creatinine) were presented as median (Q1, Q3). The p values were derived from one-way ANOVA for data consistent with a normal distribution and from Kruskal-Wallis ANOVA for data consistent with a non-normal distribution. Spearman's correlation test was used to analyze the correlation between leptin levels, sedentary time, and other cardiometabolic risk factors. We provide a scatterplot to illustrate the relationship between leptin levels and sedentary time. Additionally, we divided the participants into two groups based on their BMI: BMI < 25 and BMI ≥ 25. Then, Spearman's correlation between leptin level and sedentary time, age, FPG, TG, LDL-C, HDL-C, WC, BMI, and SBP was rerun in both groups. Finally, a linear regression analysis with sedentary time as the dependent factor was performed to evaluate the association between sedentary time and leptin levels. Three linear regression models were used. Model 1 was unadjusted; Model 2 was adjusted for age, sex, BMI, FPG, SBP, and TG; and Model 3 was adjusted for age, sex, BMI, FPG, SBP, TG, smoking, alcohol consumption, ALT, and creatinine. In our study, a *p* < 0.05 was defined as statistically significant. All statistical analyses were performed using SPSS for Windows (IBM Corp. Released 2011. IBM SPSS Statistics, version 20.0. Armonk, NY: IBM Corp.).

**Table 1:** Clinical characteristics and biochemical variables of study subjects according to tertiles of leptin level.

**Leptin**					
	**Total**	**Low**	**Middle**	**High**	
		**(**<**10.8)**	**(10.8–21.99)**	**(**≥**22.00)**	
**Variable**	***n*** = **396**	***n*** = **129**	***n*** = **136**	***n*** = **131**	***P*** **-value**
Sedentary time (hours/day)	4.83 ± 2.66	4.29 ± 2.42	4.98 ± 2.56	5.23 ± 2.90	< 0.001
Leptin (ng/mL)	8.49 (14.89, 28.36)	3.61 (6.09, 8.54)	12.57 (14.89, 17.90)	28.20 (38.32, 53.65)	< 0.001
Age (year)	64.75 ± 8.75	64.48 ± 9.44	64.00 ± 7.96	65.77 ± 8.77	0.235
FPG (mg/dl)	89.00 (99.00, 118.75)	89.00 (97.00, 111.00)	89.00 (100.00, 125.50)	91.00 (102.50, 118.00)	0.221
Triglyceride (mg/dl)	86.00 (118.00, 165.00)	81.00 (108.00, 148.50)	88.00 (125.50, 177.75)	92.75 (130.00, 168.25)	0.031
HDL-C (mg/dl)	43.00 (52.00, 61.00)	42.00 (51.00, 64.00)	43.00 (51.50, 60.75)	44.00 (53.00, 61.75)	0.438
LDL-C (mg/dl)	109.69 ± 33.99	107.85 ± 31.20	110.67 ± 32.35	110.55 ± 38.19	0.525
WC (cm)	85.36 ± 10.83	82.81 ± 9.85	84.08 ± 10.00	89.17 ± 11.58	< 0.001
BMI (kg/m2)	25.59 ± 3.84	23.73 ± 2.93	24.93 ± 2.95	28.11 ± 4.10	< 0.001
SBP (mmHg)	137.30 ± 17.49	135.38 ± 17.12	136.20 ± 16.79	140.33 ± 18.25	0.022
DBP (mmHg)	85.19 ± 10.98	84.56 ± 11.60	85.08 ± 11.25	85.92 ± 10.07	0.324
ALT (U/L)	16.00 (21.00, 30.00)	15.00 (20.00, 28.75)	16.00 (21.00, 29.75)	17.00 (22.00, 32.75)	0.235
Creatinine (mg/dl)	0.68 (0.80, 0.97)	0.72 (0.87, 1.01)	0.66 (0.78, 0.95)	0.68 (0.79, 0.96)	0.080
Gender, male (%)	164.00 (41.41%)	93.00 (70.45%)	48.00 (36.36%)	23.00 (17.42%)	0.001
Smoking (%)	50.00 (12.63%)	21.00 (15.91%)	22.00 (16.67%)	7.00 (5.30%)	< 0.001
Drinking (%)	28.00 (7.07%)	14.00 (10.61%)	11.00 (8.33%)	3.00 (2.27%)	< 0.001
HTN (%)	202.00 (50.76%)	61.00 (45.45%)	70.00 (53.03%)	71.00 (53.79%)	0.201
DM (%)	133.00 (33.59%)	44.00 (33.33%)	43.00 (32.58%)	46.00 (34.85%)	0.795
Dyslipidemia (%)	153.00 (38.64%)	43.00 (32.58%)	52.00 (39.39%)	58.00 (43.94%)	0.064

Data are expressed as the mean ± (SD) for continuous variables with a normal distribution, as the median (Q1, Q3) for continuous variables that significantly deviated from a normal distribution, and as n (%) for categorical variables.

FPG, fasting plasma glucose; HDL-C, high density lipoprotein; LDL-C, low density lipoprotein; WC, waist circumference; BMI, body mass index; SBP, systolic blood pressure; DBP, diastolic blood pressure; ALT, alanine transaminase; HTN, hypertension; DM, diabetes mellitus.

## 3. Results

This study included middle-aged and older adults from communities in northern Taiwan. Of the 396 participants, 164 were men (41.4%) and 232 were women (58.6%), with a mean age of 64.75 ± 8.75 years. The median leptin level of the study group was 8.49 (14.89, 28.36) ng/mL. The clinical characteristics, including cardiometabolic indicators, of the study, are summarized in Table 1. No statistically significant difference was found between the different leptin groups in age, FPG, HDL-C and LDL-C, DBP, ALT, or creatinine. There was no statistically significant difference in the prevalence of HTN, DM, or dyslipidemia among the three leptin groups. However, participants in the high leptin group tended to have larger waist circumferences, higher TG levels, higher BMI, and higher SBP. Participants in the high leptin group were more likely to be female and less likely to drink or smoke. Additionally, the tertiles were positively correlated with sedentary time among low, middle, and high leptin levels with statistical significance (*p* < 0.001).

**Figure 1:**
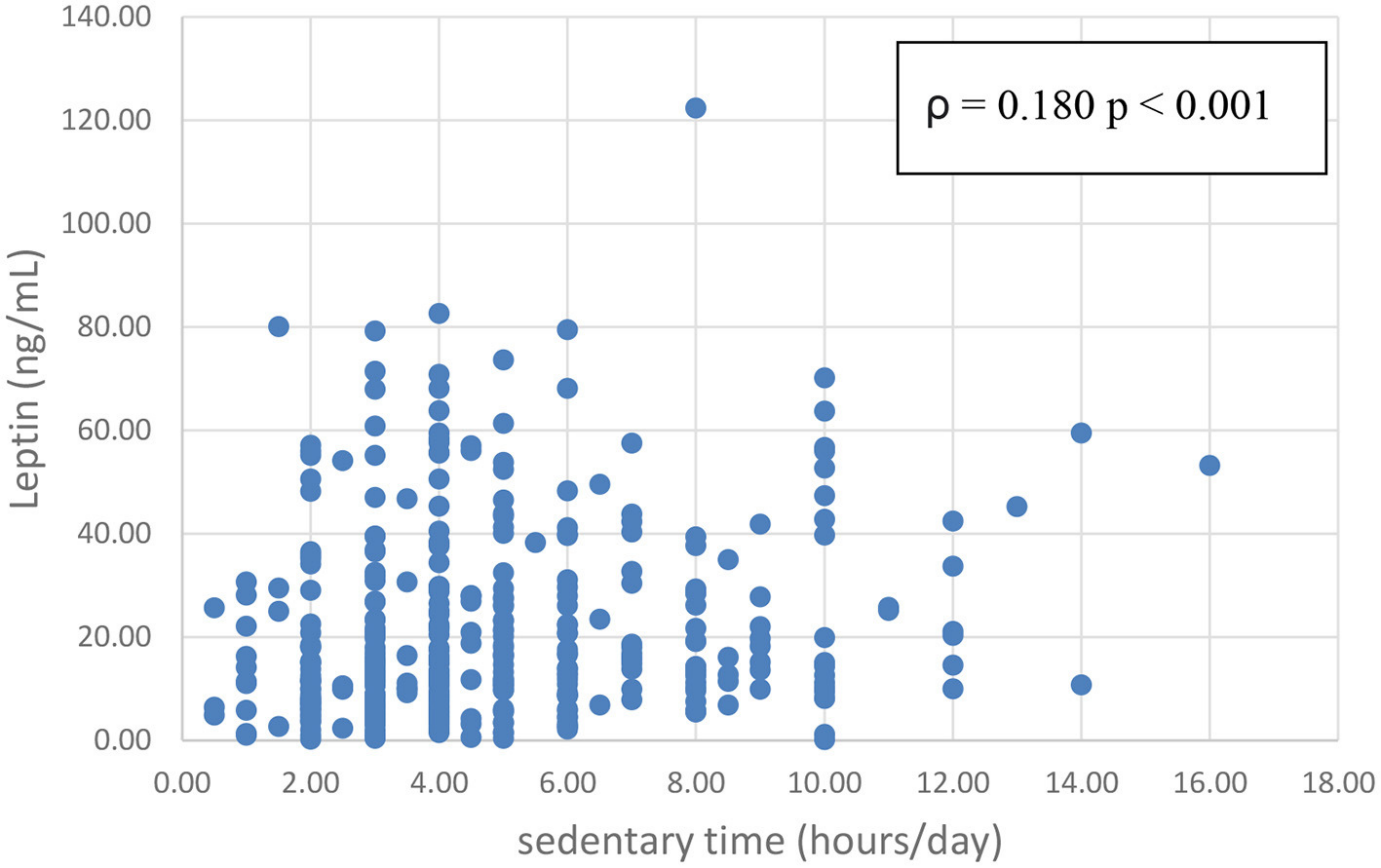
The scatterplot shows the relationship between sedentary time and leptin.

Spearman's correlation coefficient between leptin level and sedentary time was 0.151 with a *p* < 0.001. To illustrate the relationship between sedentary time and leptin level, the scatterplot of sedentary time by leptin level with the result of Spearman's correlation is shown in Figure 1. To assess the correlation between sedentary behavior and leptin, Spearman's correlation analysis was further stratified by BMI (< 25 and ≥25 kg/m^2^) to evaluate the relationship between leptin level, sedentary time, and other cardiometabolic risk factors, including age, FPG, TGs, LDL-C, HDL-C, WC, BMI, and SBP. Among all cardiometabolic risk factors, only HDL-C showed a significant correlation with leptin level in the BMI ≥25 kg/m^2^ group. However, we observed that the leptin levels had a significant positive correlation with sedentary time in both groups. Spearman's correlation coefficient between leptin level and sedentary time was 0.150 (*p* = 0.039) and 0.209 (*p* = 0.003) in the group with BMI < 25 kg/m^2^ and the group with BMI ≥25 kg/m^2^, respectively.

The results of the three linear regression models used to examine the association between leptin levels and sedentary time are shown in Table 3. Model 1 was unadjusted, and the coefficient between leptin level and sedentary time was 1.033 with a *p*-value of 0.003; Model 2 was adjusted for age, sex, BMI, FPG, SBP, and TGs, and the coefficient between leptin level and sedentary time was 0.633 with a *p*-value of 0.010; and Model 3 was adjusted for age, sex, BMI, FPG, SBP, TGs, smoking, alcohol consumption, uric acid, ALT, and creatinine, and the coefficient between leptin level and sedentary time was 0.603 with a *p*-value of 0.016. A statistically significant positive association was found between leptin level and sedentary time in all three models, and sedentary time appears to be an independent factor for leptin level.

## 4. Discussion

The initial purpose of leptin is energy balance; therefore, leptin levels are mainly influenced by physical activity, lifestyle, and body fat content (29). In Table 1, our results are consistent with those of previous studies showing the relationship between TGs, WC, BMI, SBP, smoking, alcohol consumption, and male sex. Larger WC and higher BMI are common characteristics related to obesity (30). Therefore, WC and BMI have a positive relationship with leptin levels (31, 32). Although leptin can accelerate the metabolism of TGs and decrease the secretion in the liver (33), we observed high TG levels in the high leptin group. The possible reason is that individuals with obesity, especially those with central obesity, tend to have higher TG and leptin levels (34). Those in the high leptin group tended to have higher BMI, WC and TGs. On average, women have more adipose tissue than men, which may contribute to higher plasma leptin concentrations in the female population (35). In our study, we also found that women tended to have higher leptin levels. Smoking can increase plasma catecholamine levels, and a previous study has shown that increased catecholamine levels can decrease plasma leptin levels (36). The relationship between leptin levels and alcohol consumption is inconsistent in different populations. Some studies have shown a positive relationship between alcohol consumption and leptin levels in postmenopausal women (37), but other studies have found that alcohol consumption can suppress the secretion of leptin from adipocytes and leads to low plasma leptin levels (38). In our study, we found a negative relationship between alcohol consumption and plasma leptin levels. In other studies, increased leptin levels in obese individuals were found to stimulate the dorsomedial hypothalamus and increase sympathetic nervous system activity. This mechanism might explain the positive relationship between leptin and blood pressure in obesity (39), and we also found that higher leptin levels related to higher SBP in our study. Leptin levels are closely related to physical activity (40), and most studies have emphasized the relationship between exercise and leptin levels (41, 42). In our study, we found that a sedentary lifestyle related to high leptin levels. As shown in Table 1, sedentary time was positively related to leptin levels. Based on the results in Table 1, we wanted to speculate further on the relationship between sedentary time and leptin levels.

Spearman's correlation coefficient between leptin level and sedentary time was 0.151 with a *p* < 0.001. The result is shown with a scatter plot in Figure 1. In Table 2, we divided participants into two groups based on BMI and Taiwan overwirght criteria (43). Spearman's correlation between leptin levels and sedentary time along with other cardiometabolic risk factors were evaluated. Both groups showed a significant positive correlation between leptin level and sedentary time. Our results show that whether overwight or not, sedentary time still correlates positively with leptin levels.

**Table 2:** Spearman's correlation analysis with plasma leptin level as the dependent variable in different BMI group.

**Leptin**				
	**BMI**<**25**		**BMI** ≥**25**	
**Variables**	**Correlation**	***p*** **-value**	**Correlation**	***p*** **-value**
	**coefficient (r)**		**coefficient (r)**	
Sedentary time (hours/day)	0.150	0.039	0.209	0.003
Age (year)	0.019	0.796	0.130	0.064
FPG (mg/dl)	−0.006	0.935	−0.012	0.870
Triglyceride (mg/dl)	0.118	0.104	−0.048	0.495
LDL C (mg/dl)	0.011	0.880	−0.025	0.727
HDL-C (mg/dl)	−0.021	0.777	0.279	< 0.001
WC (cm)	−0.078	0.287	0.133	0.058
SBP (mmHg)	0.024	0.740	0.087	0.214

BMI, body mass index; FPG, fasting plasma glucose; LDL-C, low density lipoprotein; HDL-C, high density lipoprotein; WC, waist circumference; SBP, systolic blood pressure.

BMI and FPG are the most important risk factors for many metabolic diseases (44). SBP, TGs, smoking and alcohol consumption are considered as risk factors for various cardiovascular diseases (45). Because we would like to discuss the relationship between leptin level and sedentary time considering cardiometabolic risk factors, we included not only the above risk factors but also other basic parameters such as age, sex, liver function (ALT), and kidney function (creatinine) in the linear regression in the models of Table 3. Incidentally, gender, BMI, SBP, TGs, smoking, alcohol consumption, ALT, and creatinine all showed a significant relationship with leptin levels in Table 1. The multiple linear regression analysis is shown in Table 3. Serum leptin levels were still positively associated with sedentary time after adjustment for age, sex, BMI, FPG, SBP, TGs, smoking, alcohol consumption, ALT, and creatinine. This result indicates that sedentary time is an independent risk factor for serum leptin levels.

**Table 3:** Multiple linear regression analysis for leptin levels in relation to sedentray time after adjustment for potential confounders.

**Model 1**				**Model 2**			**Model 3**		
	**B**	**95% CI of B**	***P*** **-value**	**B**	**95% CI of B**	***P*** **-value**	**B**	**95% CI of B**	***P*** **-value**
Sedentary time	1.033	0.364–1.702	0.003	0.633	0.149–1.117	0.010	0.603	0.120–1.086	0.016

Model 1 was unadjusted;

Model 2 was adjusted for age, gender, body mass index, fasting plasma glucose, systolic blood pressure and triglyceride;

Model 3 was adjusted for age, gender, body mass index, fasting plasma glucose, systolic blood pressure, triglyceride, smoking, drinking, alanine-transaminase, and creatinine.

B, Unstandardized regression coefficient; CI, confidence interval; S.E., standard deviation.

There are several possible explanations for the association between sedentary time and serum leptin levels. A sedentary lifestyle is closely related to obesity (46). In our study, we observed that people who sit longer tend to have a higher BMI. Obese people have excess adipocytes and produce more leptin than people with a normal BMI (47). Therefore, obesity could be the reason for the increased serum leptin in participants who sat longer in our study. In Table 2, we found a significant correlation between sedentary time and leptin levels in the obese population. However, a significant correlation between sedentary time and leptin levels was also seen in the population with normal BMI. This result suggests other mechanisms between leptin levels and sedentary time. The sympathetic tone could be another factor in the relationship between sedentary time and serum leptin levels (48). Previous studies have shown that mRNA levels and secretion of leptin in adipocyte tissues of obese people remain constant after the administration of isoproterenol (49). Activation of beta-adrenoceptors, such as the beta1-adrenoceptor and beta 2-adrenoceptor, can suppress the expression of mRNA and decrease the release of leptin (50). Exercise may increase serum catecholamine levels, which may decrease serum leptin levels, and prolonged physical activity decreases serum leptin levels (49, 51). In contrast, prolonged sedentary time means less physical activity and may lead to higher leptin levels.

Leptin may be a crucial link between a sedentary lifestyle and cardiometabolic diseases and disorders. In addition to the known link between leptin and metabolic disorders and diseases (52), leptin also affects the cardiovascular system. Leptin can stimulate the sympathetic nervous system, and elevated leptin levels can lead to high blood pressure (53), which is a risk factor for cardiovascular diseases (54). Leptin can also modulate the immune system and trigger inflammatory processes (21). Leptin may increase C-reactive protein and proinflammatory cytokines, including interleukin (IL)-1, tumor necrosis factor-α (TNF-α), and IL-6 (55, 56). These proinflammatory cytokines, in turn, cause adipocytes to secrete more leptin and proinflammatory cytokines such as TNF-α (57). The inflammation caused by leptin stimulates vascular inflammation (58) and eventually leads to cardiovascular disease (59). Recent studies also suggest that leptin can induce the accumulation of reactive oxygen species, which can activate the JNK/SAPK-dependent signaling pathway and the redox-sensitive transcription factor NF-κB (60). These signaling pathways may contribute to endothelial dysfunction in cardiovascular disease (61).

This study has many strengths. Previous studies have shown a strong relationship between leptin level and sedentary time, even independent of intense physical activity (27). However, aging is a risk factor for cardiometabolic disease (28), and there is a lack of research focused on the relationship between sedentary time and leptin levels in middle-aged and older adult populations. Therefore, we recruited community-dwelling individuals from the local clinic rather than from the participants with long-term health conditions, so our results can truly represent the situation of the middle-aged and older adult population in the community. Our study could be a reference for promoting a healthy lifestyle and preventing cardiometabolic diseases in primary care. Other strengths of our study include sufficient sample size, clear design, sufficient and relevant confounders, and rigorous data analysis. However, there are some limitations to our study. Regarding physical activity, although we recorded the intensity and duration of physical activity, many participants could not fully recall it. This shortcoming complicates the analysis of physical activity data. Accurate physical activity data and sedentary behavior data should be collected from accelerometers. Moreover, sedentary time can be more accurately recorded by accelerometers than by self-report, because there is generally a discrepancy between self-reported sedentary time and the actual sedentary time. Using personal electronic devices with accelerometers to collect data is our goal for a future study. Another obvious limitation is that our participants were all from northern Taiwan, so there may be selection bias between our results and those of the entire middle-aged and older adult population.

## 5. Conclusion

Prolonged sedentary time can be an independent risk factor for high serum leptin levels, and high leptin levels can be a novel marker in future healthcare to screen the individual with prolonged sedentary time. Furthermore, based on our study, future research can further explore the relationship between leptin levels and health promotion, especially decreasing sedentary time in the middle-aged and elder population, which is vulnerable to cardiometabolic diseases.

## Data availability statement

The raw data supporting the conclusions of this article will be made available by the authors, without undue reservation.

## Ethics statement

The studies involving human participants were reviewed and approved by Chang Gung Medical Foundation Institutional Review Board. The patients/participants provided their written informed consent to participate in this study.

## Author contributions

Y-LS composed article and conducted the study. Y-HS and T-CH helped composing article. C-CS helped data collection. J-YC provided instruction and consultation. All authors contributed to the article and approved the submitted version.

## References

[1] Vuori I. Physical inactivity is a cause and physical activity is a remedy for major public health problems. Kinesiology. (2004) 36:123–53.

[2] Klil-Drori S Cinalioglu K Rej S. The aging brain and benefits of exercise versus increased prevalence of sedentary lifestyle: lessons learned from behavioral economics on irrational decision making. Am J Geriat Psychiatry. (2022) 30:S72–3. 10.1016/j.jagp.2022.01.068

[3] Park JH Moon JH Kim HJ Kong MH Oh YH. Sedentary lifestyle: overview of updated evidence of potential health risks. Korean J Fam Med. (2020) 41:365. 10.4082/kjfm.20.0165 33242381 PMC7700832

[4] Biswas A Oh PI Faulkner GE Bajaj RR Silver MA Mitchell MS. Sedentary time and its association with risk for disease incidence, mortality, and hospitalization in adults: a systematic review and meta-analysis. Ann Intern Med. (2015) 162:123–32. 10.7326/M14-1651 25599350

[5] Caruso MV Serra R Perri P Buffone G Caliò FG Franciscis S. A computational evaluation of sedentary lifestyle effects on carotid hemodynamics and atherosclerotic events incidence. Acta Bioeng Biomech. (2017) 19:42–52. 10.5277/ABB-00682-2016-03 29205211

[6] León-Latre M Moreno-Franco B Andrés-Esteban EM Ledesma M Laclaustra M Alcalde V. Sedentary lifestyle and its relation to cardiovascular risk factors, insulin resistance and inflammatory profile. Revista Española de Cardiologí*a*. (2014) 67:449–55. 10.1016/j.rec.2013.10.015 24863593

[7] Vancampfort D Sienaert P Wyckaert S De Hert M Stubbs B. Probst M. Sitting time, physical fitness impairments and metabolic abnormalities in people with bipolar disorder: an exploratory study. Psychiatry Res. (2016) 242:7–12. 10.1016/j.psychres.2016.05.023 27235986

[8] Gruzdeva O Borodkina D Uchasova E Dyleva Y Barbarash O. Leptin resistance: underlying mechanisms and diagnosis. Diabetes Metab Syndr Obes. (2019) 12:191. 10.2147/DMSO.S182406 30774404 PMC6354688

[9] Lee IM Shiroma EJ Lobelo F Puska P Blair SN Katzmarzyk PT. Effect of physical inactivity on major non-communicable diseases worldwide: an analysis of burden of disease and life expectancy. The lancet. (2012) 380:219–29. 10.1016/S0140-6736(12)61031-9 22818936 PMC3645500

[10] Almabhouh FA Md Mokhtar AH Malik IA Aziz N Durairajanayagam D Singh HJ. Leptin and reproductive dysfunction in obese men. Andrologia. (2020) 52:e13433. 10.1111/and.13433 31773771

[11] Caron A Lee S Elmquist JK Gautron L. Leptin and brain–adipose crosstalks. Nat Rev Neurosci. (2018) 19:153–65. 10.1038/nrn.2018.7 29449715 PMC5962962

[12] Fruhwürth S Vogel H Schürmann A Williams KJ. Novel insights into how overnutrition disrupts the hypothalamic actions of leptin. Front Endocrinol. (2018) 9:89. 10.3389/fendo.2018.00089 29632515 PMC5879088

[13] Popovic V Duntas LH. Leptin TRH and ghrelin: influence on energy homeostasis at rest and during exercise. Horm Metab Res. (2005) 37:533–7. 10.1055/s-2005-870418 16175489

[14] Barrios V Frago LM Canelles S Guerra-Cantera S Arilla-Ferreiro E Chowen JA Argente J. Leptin modulates the response of brown adipose tissue to negative energy balance: implication of the GH/IGF-I axis. Int J Mol Sci. (2021) 22:2827. 10.3390/ijms22062827 33799501 PMC8001882

[15] Thon M Hosoi T Ozawa K. Possible integrative actions of leptin and insulin signaling in the hypothalamus targeting energy homeostasis. Front Endo. (2016) 7:138. 10.3389/fendo.2016.00138 27812350 PMC5071376

[16] Suriagandhi V Nachiappan V. Protective effects of melatonin against obesity-induced by leptin resistance. Behav Brain Res. (2022) 417:113598. 10.1016/j.bbr.2021.113598 34563600

[17] López-Jaramillo P Gómez-Arbeláez D López-López J López-López C Martínez-Ortega J Gómez-Rodríguez A. The role of leptin/adiponectin ratio in metabolic syndrome and diabetes. Horm Mol Biol Clin Investig. (2014) 18:37–45. 10.1515/hmbci-2013-0053 25389999

[18] Shankar A Xiao J. Positive relationship between plasma leptin level and hypertension. Hypertension. (2010) 56:623–8. 10.1161/HYPERTENSIONAHA.109.148213 20713919

[19] Tsai JP. The association of serum leptin levels with metabolic diseases. Tzu-Chi Med J. (2017) 29:192. 10.4103/tcmj.tcmj_123_17 29296046 PMC5740690

[20] Landry D Cloutier F Martin LJ. Implications of leptin in neuroendocrine regulation of male reproduction. Reproductive biology. (2013) 13:1–14. 10.1016/j.repbio.2012.12.001 23522066

[21] Pérez-Pérez A Vilariño-García T Fernández-Riejos P Martín-González J Segura-Egea JJ Sánchez-Margalet V. Role of leptin as a link between metabolism and the immune system. Cytokine Growth Factor Rev. (2017) 35:71–84. 10.1016/j.cytogfr.2017.03.001 28285098

[22] Katsiki N Mikhailidis DP Banach M. Leptin, cardiovascular diseases and type 2 diabetes mellitus. Acta Pharmacol Sin. (2018) 39:1176–88. 10.1038/aps.2018.40 29877321 PMC6289384

[23] Mao S Fang L Liu F Jiang S Wu L Zhang J. Leptin and chronic kidney diseases. J Recep Signal Trans. (2018) 38:89–94. 10.1080/10799893.2018.1431278 29388492

[24] Zou X Zhong L Zhu C Zhao H Zhao F Cui R. Role of leptin in mood disorder and neurodegenerative disease. Front Neurosci. (2019) 13:378. 10.3389/fnins.2019.00378 31130833 PMC6510114

[25] Patterson R McNamara E Tainio M de Sá TH Smith AD Sharp SJ. Sedentary behaviour and risk of all-cause, cardiovascular and cancer mortality, and incident type 2 diabetes: a systematic review and dose response meta-analysis. Eur J Epidemiol. (2018) 33:811–29. 10.1007/s10654-018-0380-1 29589226 PMC6133005

[26] Allison MA Jensky NE Marshall SJ Bertoni AG Cushman M. Sedentary behavior and adiposity-associated inflammation: the multi-ethnic study of atherosclerosis. Am J Prev Med. (2012) 42:8–13. 10.1016/j.amepre.2011.09.023 22176840 PMC3244676

[27] Henson J Yates T Edwardson CL Khunti K Talbot D Gray LJ. Sedentary time and markers of chronic low-grade inflammation in a high risk population. PLoS ONE. (2013) 8:e78350. 10.1371/journal.pone.0078350 24205208 PMC3812126

[28] Das A Reis F Maejima Y Cai Z Ren J. mTOR signaling in cardiometabolic disease, cancer, and aging. Oxid Med Cell Longev. (2017) 2017:6018675. 10.1155/2017/6018675 28770023 PMC5523348

[29] Herrick JE Panza GS Gollie JM. Leptin, leptin soluble receptor, and the free leptin index following a diet and physical activity lifestyle intervention in obese males and females. J Obes. (2016) 2016:8375828. 10.1155/2016/8375828 28050279 PMC5168550

[30] Nuttall FQ. Body mass index: obesity. BMI, and health: a critical review. Nutr Today. (2015) 50:117. 10.1097/NT.0000000000000092 27340299 PMC4890841

[31] Al Maskari MY Alnaqdy AA. Correlation between serum leptin levels, body mass index and obesity in Omanis. Sultan Qaboos Univ Med J. (2006) 6:27–31. 21748132 PMC3074914

[32] Zoccali C Postorino M Marino C Pizzini P Cutrupi S Tripepi G. Waist circumference modifies the relationship between the adipose tissue cytokines leptin and adiponectin and all-cause and cardiovascular mortality in haemodialysis patients. J Int Med. (2011) 269:172–81. 10.1111/j.1365-2796.2010.02288.x 21138492

[33] Huang W Dedousis N Bandi A Lopaschuk GD O'Doherty RM. Liver triglyceride secretion and lipid oxidative metabolism are rapidly altered by leptin *in vivo*. Endocrinology. (2006) 147:1480–7. 10.1210/en.2005-0731 16339207

[34] Wang K He G Zhang Y Yin J Yan Y Zhang Y. Association of triglyceride-glucose index and its interaction with obesity on hypertension risk in Chinese: a population-based study. J Hum Hypertens. (2021) 35:232–9. 10.1038/s41371-020-0326-4 32203074

[35] Hellström L Wahrenberg H Hruska K Reynisdottir S Arner P. Mechanisms behind gender differences in circulating leptin levels. J Intern Med. (2000) 247:457–62. 10.1046/j.1365-2796.2000.00678.x 10792559

[36] Reseland JE Mundal HH Hollung K Haugen F Zahid N Anderssen SA. Cigarette smoking may reduce plasma leptin concentration via catecholamines. Prostaglandins Leukot Essent Fatty Acids. (2005) 73:43–9. 10.1016/j.plefa.2005.04.006 15964536

[37] Roth MJ Baer DJ Albert PS Castonguay TW Dorgan JF Dawsey SM. Relationship between serum leptin levels and alcohol consumption in a controlled feeding and alcohol ingestion study. J Natl Cancer Inst. (2003) 95:1722–5. 10.1093/jnci/djg090 14625264

[38] Otaka M Konishi N Odashima M Jin M Wada I Matsuhashi T. Effect of alcohol consumption on leptin level in serum, adipose tissue, and gastric mucosa. Dig Dis Sci. (2007) 52:3066–9. 10.1007/s10620-006-9635-x 17406835

[39] Enriori PJ Sinnayah P Simonds SE Rudaz CG Cowley MA. Leptin action in the dorsomedial hypothalamus increases sympathetic tone to brown adipose tissue in spite of systemic leptin resistance. J Neurosci. (2011) 31:12189–97. 10.1523/JNEUROSCI.2336-11.2011 21865462 PMC3758545

[40] Haluzikova D Haluzik M Nedvidkova J Boudova L Brandejský P Barackova M. Effect of physical activity on serum leptin levels. Sbornik Lekarsky. (2000) 101:89–92. 10953637

[41] Gomez-Merino D Chennaoui M Drogou C Bonneau D Guezennec CY. Decrease in serum leptin after prolonged physical activity in men. Med Sci Sports Exerc. (2002) 34:1594–9. 10.1097/00005768-200210000-00010 12370560

[42] Romon M Lafay L Bresson JL Oppert JM Borys JM Kettaneh A. Relationships between physical activity and plasma leptin levels in healthy children: the Fleurbaix–Laventie Ville Sante II Study. Int J Obes. (2004) 28:1227–32. 10.1038/sj.ijo.0802725 15314633

[43] Chu NF. Prevalence of obesity in Taiwan. Obes Rev. (2005) 6:271–4. 10.1111/j.1467-789X.2005.00175.x 16246212

[44] Han TS Lean ME. A clinical perspective of obesity, metabolic syndrome and cardiovascular disease. JRSM Cardiovasc Dis. (2016) 5. 10.1177/2048004016633371 26998259 PMC4780070

[45] Foerster M Marques-Vidal P Gmel G Daeppen JB Cornuz J Hayoz D. Alcohol drinking and cardiovascular risk in a population with high mean alcohol consumption. Am J Cardiol. (2009) 103:361–8. 10.1016/j.amjcard.2008.09.089 19166690

[46] Silveira EA Mendonça CR Delpino FM Souza GVE de Souza Rosa LP de Oliveira C. sedentary behavior, physical inactivity, abdominal obesity and obesity in adults and older adults: A systematic review and meta-analysis. Clin Nutr ESPEN. (2022) 50:63–73. 10.1016/j.clnesp.2022.06.001 35871953

[47] Obradovic M Sudar-Milovanovic E Soskic S Essack M Arya S Stewart AJ. Leptin and obesity: role and clinical implication. Front Endocrinol. (2021) 12:585887. 10.3389/fendo.2021.585887 34084149 PMC8167040

[48] Littman AJ Vitiello MV Foster-Schubert K Ulrich CM Tworoger SS Potter JD. Sleep, ghrelin, leptin and changes in body weight during a 1-year moderate-intensity physical activity intervention. Int J Obes. (2007) 31:466–75. 10.1038/sj.ijo.0803438 16909130

[49] Eikelis N Schlaich M Aggarwal A Kaye D Esler M. Interactions between leptin and the human sympathetic nervous system. Hypertension. (2003) 41:1072–9. 10.1161/01.HYP.0000066289.17754.49 12668587

[50] Ricci MR Lee MJ Russell CD Wang Y Sullivan S Schneider SH. Isoproterenol decreases leptin release from rat and human adipose tissue through posttranscriptional mechanisms. Am J Physiol Endocrinol Metab. (2005) 288:E798–804. 10.1152/ajpendo.00446.2004 15585586

[51] Zaccaria M Ermolao A Brugin E Bergamin M. Plasma leptin and energy expenditure during prolonged, moderate intensity, treadmill exercise. J Endocrinol Invest. (2013) 36:396–401. 10.3275/8656 23095336

[52] Wróblewski A Strycharz J Swiderska E Drewniak K Drzewoski J Szemraj J. Molecular insight into the interaction between epigenetics and leptin in metabolic disorders. Nutrients. (2019) 11:1872. 10.3390/nu11081872 31408957 PMC6723573

[53] Rahmouni K. Leptin-induced sympathetic nerve activation: signaling mechanisms and cardiovascular consequences in obesity. Curr Hypertens Rev. (2010) 6:104–9. 10.2174/157340210791170994 21562617 PMC3090157

[54] Fuchs FD Whelton PK. High blood pressure and cardiovascular disease. Hypertension. (2020) 75:285–92. 10.1161/HYPERTENSIONAHA.119.14240 31865786 PMC10243231

[55] Rehman KA. Leptin: a new therapeutic target for treatment of diabetes mellitus. J Cell Biochem. (2018) 119:5016–27. 10.1002/jcb.26580 29236298

[56] Soni AC. Ghrelin, leptin, adiponectin, and insulin levels and concurrent and future weight change in overweight postmenopausal women. Menopause. (2011) 18:296. 10.1097/gme.0b013e3181f2e611 21449093 PMC3069721

[57] Van Gaal LF. Mechanisms linking obesity with cardiovascular disease. Nature. (2006) 444:875–80. 10.1038/nature05487 17167476

[58] Dubey L Hesong Z. Role of leptin in atherogenesis. Exp Clin Cardiol. (2006) 11:269. 18651016 PMC2274849

[59] Hou N Luo JD. Leptin and cardiovascular diseases. Clin Exp Pharmacol Physiol. (2011) 38:905–13. 10.1111/j.1440-1681.2011.05619.x 21957899

[60] Zhang BX. Renal thrombotic microangiopathies induced by severe hypertension. Hypert Res. (2008) 31:479–83. 10.1291/hypres.31.479 18497467

[61] Poetsch MS Strano A. Guan K. Role of leptin in cardiovascular diseases. Front Endocrinol. (2020) 11:354. 10.3389/fendo.2020.00354 32655492 PMC7325922

